# Effects of dexmedetomidine combined with etomidate on postoperative cognitive function in older patients undergoing total intravenous anaesthesia: a randomized, double-blind, controlled trial

**DOI:** 10.1186/s12877-024-04726-7

**Published:** 2024-01-25

**Authors:** Wuchang Fu, Hongchun Xu, Ting Zhao, Jun Xu, Fangjun Wang

**Affiliations:** 1 The Second Clinical Medical college of North, Sichuan Medical College (Nanchong Central Hospital), Nanchong, 637000 China; 2 The Department of Anesthesiology, Shunqing District, Affiliated Hospital, North Sichuan Medical College, Sichuan Province, No. 1, MaoYuan South Road, Nanchong City, 637000 China; 3 The north sichuan medical college, Nanchong, 637000 China

**Keywords:** etomidate, Dexmedetomidine, Postoperative cognitive dysfunction, Depression, Anxiety, Older patients

## Abstract

**Background:**

Etomidate has been advocated for anesthesia in older and critically ill patients because of its hemodynamic stability. Clinical studies have shown that dexmedetomidine has neuroprotective and anti-inflammatory properties and improves postoperative cognitive dysfunction in older patients. The present study was to evaluate the effects of the combination of etomidate and dexmedetomidine with different anaesthesia time on postoperative cognitive function in older patients.

**Methods:**

A total of 132 older patients undergoing ureteroscopic holmium laser lithotripsy were randomly divided into EN group and ED group equally. Patients whose surgery time was less than or equal to 1 h in each group were allocated to short-time surgery group (EN_1_ group and ED_1_ group), and whose surgery time was more than 1h were allocated to long-term surgery group (EN_2_ group and ED_2_ group). The primary outcome was the score of the Mini-Mental State Examination. The secondary outcomes were State-Trait Anxiety Inventory scores, Riker sedation agitation scores, Zung Self-Rating Depression Scale scores, the memory span for Arabic numerals, the plasma concentrations of S-100 calcium-binding protein B and neuron specific enolase, the time to spontaneous respiration, recovery, and extubation.

**Results:**

The MMSE scores at t_2-3_ were higher in ED_1_ and ED_2_ groups than in EN_1_ and EN_2_ groups (*p*<0.05). Compared with ED_1_ and ED_2_ groups, the ZSDS scores, the S-AI scores and the T-AI scores at t_1-2_ were higher in EN_1_ and EN_2_ groups (*p*<0.05), respectively. The recalled Arabic numbers at t_1-3_ were higher in ED_2_ group than in EN_2_ group (*p*<0.05). The plasma concentration of S-100β at t_1-2_ in EN_1_ group and t_1-3_ in EN_2_ group were higher than that in ED_1_ and ED_2_ groups (*p*<0.05), respectively. Compared with ED_1_ and ED_2_ groups, the plasma concentrations of NSE were higher at t_1-3_ in EN_1_ group and t_1-4_ in EN_2_ group (*p*<0.05), respectively.

**Conclusion:**

The administration of dexmedetomidine could improve postoperative cognitive dysfunction, emergence agitation, depression and anxiety, attenuate the plasma concentrations of S-100β and NSE in older patients undergoing total intravenous anaesthesia with etomidate.

**Trial registration:**

Registration number: ChiCTR1800015421, Date: 29/03/2018.

## Introduction

The surgery for the older patients is increasing with the development of the global aging society. Postoperative central nervous system complications in the older people have accumulated attention in clinical, especially cognitive dysfunction after anaesthesia in the older patients [1]. Etomidate is a non-barbiturate short-acting intravenous general anaesthetic derived from imidazole, which has good sedative effect and no analgesic effect [2]. Because of its hemodynamic stability, etomidate has been advocated clinically for anaesthesia in older and critically ill patients. The animal study reported by Zurek AA et al. showed that a single in vivo treatment with etomidate increased the tonic inhibitory current produced by α_5_ subunit-containing GABAAR (α_5_GABAAR) and the cell surface expression of α_5_GABA for nearly one week, and synaptic plasticity and memory performance in the hippocampus were impaired by the sustained increase in α_5_GABAAR activity [3]. It is suggested that the administration of etomidate can lead to neurological disorder.

Dexmedetomidine is an efficient, selective α_2_ adrenergic receptor agonist with dose-dependent sedation, analgesia, anti anxiety and sympathetic inhibition, which has few side effects and superiority and application value in clinical practice [4]. Clinical studies have shown that dexmedetomidine has neuroprotective and anti-inflammatory properties and improves postoperative cognitive dysfunction in older patients [5–7]. In previous clinical study, we found that the combination of dexmedetomidine and etomidate could maintain perioperative hemodynamic stability and reduce the inhibitory effect of etomidate on adrenocortical function in older patients [8]. However, there is no report on the effect of the combination of etomidate and dexmedetomidine for anaesthesia on postoperative cognitive function of older patients, and it is not clear whether the combination of etomidate and dexmedetomidine with different anaesthesia time has different effects on postoperative cognitive function of older patients. The purpose of present study was to evaluate the effects of the combination of etomidate and dexmedetomidine with different anaesthesia time on postoperative cognitive function in older patients.

## Methods

After obtaining the Ethics Committee of the affiliated hospital of north sichuan medical college approval in March 2018, the study was registered at the Chinese Clinical Trial Registry (http://www.chictr.org.cn/.; Registration number: ChiCTR1800015421, date: 29/03/2018), and conducted in accordance with the principles expressed in the Declaration of Helsinki and the guidelines of Good Clinical Practice. A total of 140 patients scheduled for ureteroscopic holmium laser lithotripsy between April 2018 and March 2019 were enrolled in this study. All participants signed an informed consent for participation. Patients were included if they were between 60 and 80 years old and had an American Society of Anesthesiologists status of I or II. The exclusion criteria included the following: a preoperative history of chronic use of sedatives, opioids, and alcohol; history of adverse responses to dexmedetomidine, etomidate or remifentanil; ischemic cerebrovascular disease; the presence of liver or kidney dysfunctions; cardiovascular disease such as severe arrhythmia, severe valvular heart disease, severe hypertension, coronary heart disease, heart failure, and arterial dissection; endocrine disease; smoking within 2 weeks; cognitive dysfunction; systolic blood pressure (SBP) ≥ 160 mmHg or diastolic blood pressure (DBP) ≥ 110 mmHg; BMI <18.5kg/m^2^ or >24kg/m^2^; sinus bradycardia; change in surgical plan. Withdrawal criteria: change of surgical plan, patients or relatives refusing to continue the study, and incomplete data collection.

Patients were assigned randomly to two groups. EN group received intravenous anaesthesia with etomidate-remifentanil combined with equal volume of normal saline and ED group received intravenous anaesthesia with etomidate-remifentanil combined with dexmedetomidine. Randomization was done by an anaesthetic nurse who did not participate in the trial using the random number table. After being randomly selected from the random number table, 140 three-digit numbers were serialized from small to large. The anaesthetic nurse set the serial numbers 1–70 as EN group and 71–140 as ED group and sealed all cards identifying patient grouping information in opaque envelopes. When the patient arrived in the operating room, a sealed envelope was randomly selected and the dexmedetomidine or saline was prepared according to the concealed envelope for random allocation. Patients with surgery time less than or equal to 1h in each group were included in the short-term surgery group ( EN_1_ group and ED_1_ group ) and patients with surgery time greater than 1h were included in the long-term surgery group ( EN_2_ group and ED_2_ group ). The anesthetists, nurses, surgeons, and participating patients were blinded to the treatment allocation. The primary outcome of this study was the score of the Mini-Mental State Examination (MMSE). The secondary outcomes were State-Trait Anxiety Inventory scores (STAI), Riker sedation agitation scores(RSAS), Zung Self-Rating Depression Scale scores (ZSDS), the memory span for Arabic numerals (MSAM), the plasma concentration of S-100 calcium-binding protein B (S100β), the time to spontaneous respiration, recovery, and extubation, the plasma concentration of neuron specific enolase (NSE) and the duration of surgery.

Patients were interviewed the day before the surgery and all remembered five random Arabic numbers and were familiar with questionnaires, including mini-mental state examination, the Zung self-rating depression scale and the state-trait anxiety questionnaire. All patients had no premedication prior to induction. Non-invasive blood pressure (NBP), HR, Electrocardiogram (ECG), the bispectral index, temperature and SpO_2_ were monitored once the enrolled patients entered the operating room at 3-min intervals. Induction in EN and ED groups was performed with intravenous midazolam 0.04 mg/kg, etomidate 0.4mg/kg, remifentanil 2μg/kg and cisatracurium 0.6 mg/kg. Anaesthesia in EN group was maintained with a plasma target concentration of remifentanil 4~6 ng/ml in the Minto model and etomidate 4~6μg/ml in the Arden model by TCI (TCI-III-B Infusion Pump, Guangxi Willy Ark Technology Co., Ltd., China) and intravenous infusion equal volume normal saline, while anaesthesia in ED group was maintained with a plasma target concentration of remifentanil 4~6 ng/ml in the Minto model, etomidate 4~6μg/ml in the Arden model by TCI and intravenous infusion of dexmedetomidine 0.4μg·kg^-1^·h^-1^. The intraoperative BIS values in EN and ED groups were maintained between 40 and 50. Muscle relaxant was administered intraoperatively if required. The administration of muscle relaxant was stopped 45min before the end of the surgery. Dexmedetomidine or placebo and etomidate -remifentanil were stopped 20 and 5 min before the end of the surgery, respectively. If SBP below 90 mmHg and heart rate lower than 50 beats/min, ephedrine 5mg and atropine 0.5 mg were administered, respectively. When BIS > 80, tidal volume > 6 ml/kg, a train-of-four (TOF) ratio ≥ 0.9, SpO2 > 90% under air inspiration and respiratory rate > 13/min, postoperative extubation was performed. Patients were transferred to the PACU after extubation and transferred to the surgical ward when the modified Aldrete score was >9.

The PACU emergence agitation scores at 5min, 15 min, 30 min and 60min after extubation were evaluated by an anesthesiologist blinded to the study using the Riker sedation agitation scores (RSAS: 1, unable to be awakened; 2, calmness; 3 relative calmness; 4, calm cooperation; 5, agitation; 6, great agitation; 7, extreme agitation) [9]. The Mini-Mental State Examination, State-Trait Anxiety Inventory scores, Zung Self-Rating Depression Scale scores and memory span for Arabic numerals were applied 30min before anaesthesia ( t_0_ ), 3h ( t_1_ ), 8h ( t_2_ ), 24h ( t_3_ ), 48h ( t_4_ ) and 72h ( t_5_ ) after operation. The SBP, DBP and HR were recorded 5 min before anaesthesia induction ( T_0_ ), 5 min after anaesthesia induction ( T_1_ ), at the beginning of operation ( T_2_ ), during operation ( T_3_ ), 3 h ( T_4_ ), 6 h ( T_5_ ), 24 h ( T_6_ ) and 48 h ( T_7_ ) after operation. The arousal time, time to spontaneous respiration and extubation (time from stopping the administration of etomidate-remifentanil to arousal, spontaneous respiration and extubation, respectively.) and duration of surgery were recorded. 4ml venous blood sample was taken at t_0-5_ for detection of NSE and S100β by ELISA. The dosages of etomidate and remifentanil during surgery were recorded.

## Statistical analysis

We calculated that a sample size of 28 patients was required in each group at a power of 90%, with a two-sided significance level of 0.05 by one-way analysis of varianc F-Tests for determination of a difference in MMSE scores between groups at 8h after the surgery, depending on our preliminary study results of 40 patients (The MMSE scores were 22.1±2.9, 20.6±2.5, 23.6±2.6 and 22.1±1.8 in EN_1_, ED_1_, EN_2_ and ED_2_ groups, respectively). To account for a 20% dropout rate, we included 35 patients in each group.

SPSS 22.0 program was used for statistical analyses. Normally distributed data were expresses as mean ± standard deviation. The data of age, weight, arousal time, time to spontaneous respiration and extubation, duration of surgery and dosages of etomidate and remifentanil were compared using one-way analysis of variance. The data of SBP, DBP, HR, SPO2, MMSE scores, STAI scores, ZSDS scores, MSAM scores and Plasma concentration of S100β and NSE were compared using two-way ANOVA, followed by post hoc tests. The *X*^2^ or Fisher’s exact tests was used for comparisons of the levels of education, ASA physical status, sex ratio and RSAS scores between groups. *P* < 0.05 was considered statistically significant.

## Results

**Fig.1:**
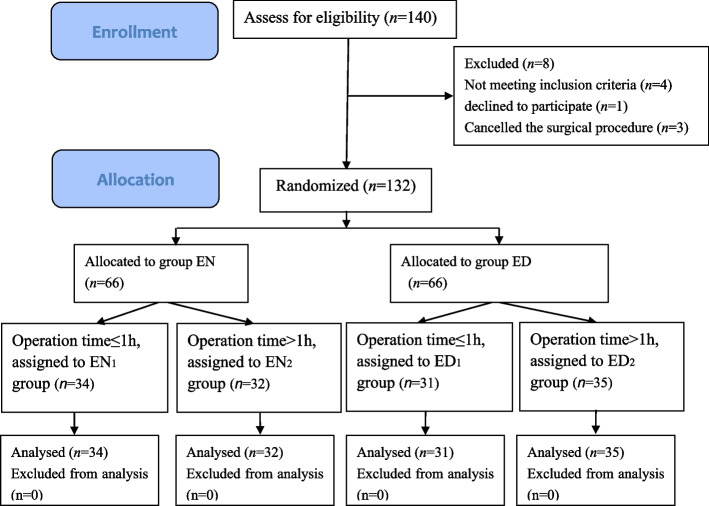
Study flow diagram

One hundred and forty patients were screened for eligibility, four patients were excluded for not meeting the inclusion criteria, three patients were cancelled the surgical procedure and one patient declined to participate. A total of 132 patients were subsequently allocated to the two groups. Thirty four and thirty one patients with surgery time less than or equal to 1h were in EN and ED groups, respectively. A total of 132 patients completed the study and were analyzed (Fig. 1).

The Demographic data in four groups are shown in Table 1. There were no differences in Demographic data between groups.

**Table 1:** Demographic data

Indexes	EN_1_ group *n*=34	ED_1_ group *n*=31	EN_2_ group *n*=32	ED_2_ group *n*=35	*F*/*X*^2^ values	*P* values
Age(year)	67.1±4.0	67.21±5.0	66.7±3.9	66.9±4.1	0.139	0.936
Weight(kg)	57.1±6.7	58.01±5.5	56.6±9.3	57.6±8.5	0.187	0.905
ASA(I/II)	14/20	14/17	14/18	15/20	0.111	0.990
BMI(kg/m^2^)	21.8±1.4	21.7±1.5	21.5±1.7	21.3±1.7	0.684	0.563
Gender(male/female)	18/16	16/15	17/15	19/16	0.293	0.961
Level of education (illiterate/primary school literacy/middle school)	13/16/5	13/14/4	14/15/3	15/15/5	0.675	0.995

*ASA* American Society of Anesthesiologists, *BMI* Body mass index, *EN*_*1*_ Etomidate-remifentanil with duration of surgery less than 60min, *ED*_*1*_ Etomidate-remifentanil and dexmedetomidine with duration of surgery less than 60min, *EN*_*2*_ Etomidate-remifentanil with duration of surgery more than 60min, *ED*_*2*_ Etomidate-remifentanil and dexmedetomidine with duration of surgery more than 60min

The clinical characteristics in four groups are shown in Table 2. The time to spontaneous respiration, time to recovery, tracheal extubation time and PACU stay time were longer in ED_1_ and ED_2_ groups compared to EN_1_ and EN_2_ groups (*p* < 0.05), respectively. Both the dosages of etomidate and remifentanil were decreased more significantly in ED_1_ and ED_2_ groups than in EN_1_ and EN_2_ groups (*p* < 0.05).

**Table 2:** Clinical characteristics

Clinical characteristics	EN_1_ group *n*=34	ED_1_ group *n*=31	EN_2_ group *n*=32	ED_2_ group *n*=35	*F* value	*P* value
Time to spontaneous respiration (min)	17.9±2.4	20.0±3.3^*^	18.0±3.0	21.0±3.2^#^	9.896	0.000
Time to recovery (min)	19.8±2.6	24.0±4.6^*^	20.1±3.5	25.1±3.5^#^	19.166	0.000
Tracheal extubation time (min)	21.6±2.8	27.0±5.5^*^	22.0±4.3	28.1±4.6^#^	18.816	0.000
PACU stay time (min)	59.0±5.3	63.0±9.6^*^	71.3±9.8	75.8±10.7^#^	26.500	0.000
Duration of surgery (min)	47.3±6.4	46.9±6.1	101.3±6.4	100.6±6.4	154.334	0.000
Dosage of etomidate (mg)	55.0±6.5	45.9±6.0^*^	93.5±11.3	75.3±7.3^#^	224.868	0.000
Dosage of remifentanil (µg)	909.8±45.9	886.9±39.2^*^	1656.9±196.3	1486.3±128.9^#^	341.445	0.000

*PACU* Postanesthesia care unit, *EN*_*1*_ Etomidate-remifentanil with duration of surgery less than 60min, *ED*_*1*_ Etomidate-remifentanil and dexmedetomidine with duration of surgery less than 60min, EN_2_ Etomidate-remifentanil with duration of surgery more than 60min, *ED*_*2*_ Etomidate-remifentanil and dexmedetomidine with duration of surgery more than 60min

^*^ *p* < 0.05 vs.EN1, ^#^ *p* < 0.05 vs.EN2.

**Fig 2:**
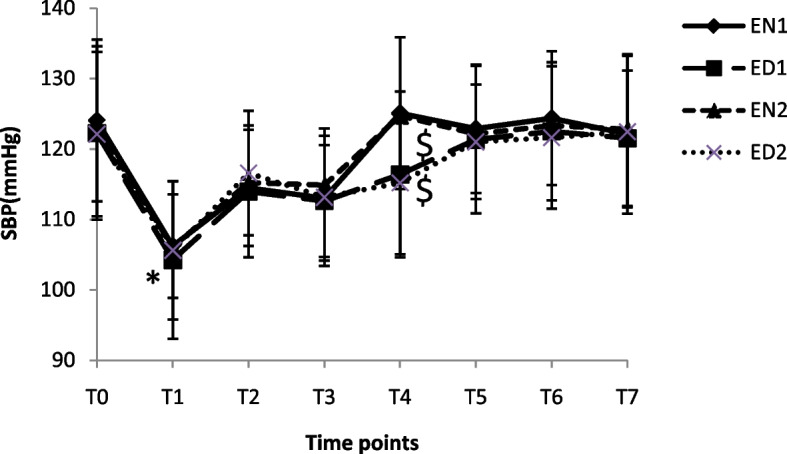
The SBP at different time points in four groups. *p < 0.05 vs.T_0_, ^#^ *p* < 0.05 vs.EN_1_ and ^$^ *p* < 0.05 vs.EN_2_

The SBP at different time points in four groups is shown in Fig 2. Compared to T_0_, the SBP was decreased more significantly at T_1-3_ in EN_1_ and EN_2_ groups and T_1-4_ in ED_1_ and ED_2_ groups (*p* < 0.05). At T_4_, the SBP in EN_1_ and EN_2_ groups was higher than that in ED_1_ and ED_2_ groups, respectively (*p* < 0.05).

**Fig 3:**
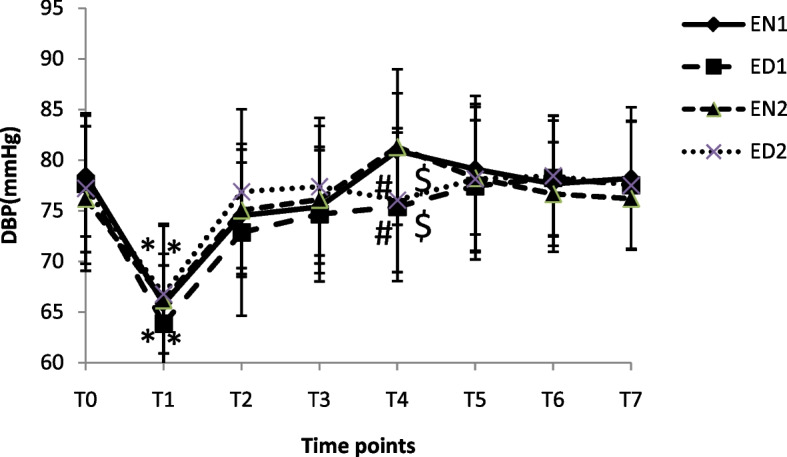
The DBP a different time points in four groups. *p < 0.05 vs.T_0_, ^#^ *p* < 0.05 vs.EN_1_ and ^$^ *p* < 0.05 vs.EN_2_

The DBP at different time points in four groups is shown in Fig 3. The DBP in four groups were lower at T_1_ compared to T_0_ (*p* < 0.05). The DBP at T_4_ in EN_1_ and EN_2_ groups were significantly higher than that in ED_1_ and ED_2_ groups (*p* < 0.05).

**Fig 4:**
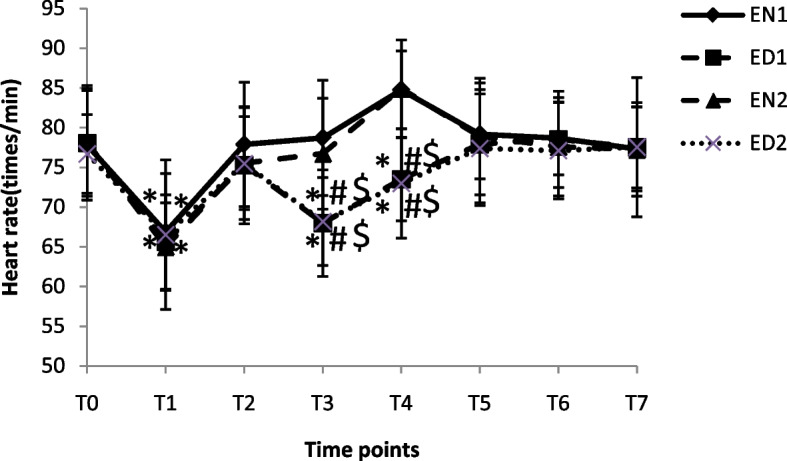
The HR at different time points in four groups. * *p* < 0.05 vs.T_0_, ^#^ *p* < 0.05 vs. EN_1_ and ^$^ *p* < 0.05 vs. EN_2_

The HR at different time points in four groups is shown in Fig 4. Compared to T_0_, the HR at T_1_ in four groups and at T_3-4_ in ED_1_ and ED_2_ groups were decreased more significantly (*p* < 0.05). The HR at T_3-4_ in EN_1_ and EN_2_ groups were significantly higher than that in EN1 and EN2 groups (*p* < 0.05), respectively.

The RSAS scores after extubation in the four groups are shown in Table 3. The RSAS scores at 5, 15, 30 and 60min after extubation were higher in EN_1_ and EN_2_ groups than in ED_1_ and ED_2_ groups (*p* < 0.05).

**Table 3:** The Riker sedation agitation scores after extubation in four groups

Time points	Groups	n	Riker sedation agitation scores						
			1	2	3	4	5	6	7
5min after extubation	EN_1_	34	0	0	2	7	13	10	2
	ED_1_^*^	31	0	2	9	15	4	1	0
	EN_2_	32	0	0	2	6	13	8	3
	ED_2_^#^	35	0	1	7	16	9	2	0
	*X*^2^ value	41.228							
	*P* value	0.000							
15min after extubation	EN_1_	34	0	2	4	6	12	9	1
	ED_1_^*^	31	0	6	13	9	3	0	0
	EN_2_	32	0	0	0	7	13	9	3
	ED_2_^#^	35	0	8	8	12	7	0	0
	*X*^2^ value	59.285							
	*P* value	0.000							
30min after extubation	EN_1_	34	0	2	4	9	12	7	0
	ED_1_^*^	31	0	10	14	6	1	0	0
	EN_2_	32	0	0	0	8	14	8	2
	ED_2_^#^	35	0	6	10	13	6	0	0
	*X*^2^ value	67.804							
	*P* value	0.000							
60min after extubation	EN_1_	34	0	0	2	13	14	5	0
	ED_1_^*^	31	0	2	16	12	1	0	0
	EN_2_	32	0	0	0	13	12	6	1
	ED_2_^#^	35	0	4	10	16	5	0	0
	*X*^2^ value	60.015							
	*P* value	0.000							

*EN*_*1*_ Etomidate-remifentanil with duration of surgery less than 60min, *ED*_*1*_ Etomidate-remifentanil and dexmedetomidine with duration of surgery less than 60min, *EN*_*2*_ Etomidate-remifentanil with duration of surgery more than 60min, *ED*_*2*_ Etomidate-remifentanil and dexmedetomidine with duration of surgery more than 60min

^*^ *p* < 0.05 vs.EN_1_, ^#^ *p* < 0.05 vs.EN_2_

The MMSE scores at different time points in four groups are shown in Table 4. The MMSE scores at t_1-3_ in EN_1_, EN_2_ and ED_2_ groups and at t_1-2_ in ED_1_ group were decreased more significantly compared to t_0_ (*p* < 0.05). The MMSE scores at t_2-3_ were higher in ED_1_ and ED_2_ groups than in EN_1_ and EN_2_ groups (*p* < 0.05), respectively. The MMSE scores at t_3_ were lower in EN_2_ and ED_2_ groups than in EN_1_ and ED_1_ groups (*p* < 0.05), respectively.

**Table 4:** The MMSE scores at different time points in four groups

Time points	EN_1_ group *n*=34	ED_1_ group *n*=31	EN_2_ group *n*=32	ED_2_ group *n*=35	*F* values	*P* values
t_0_	25.8±1.5	25.7±1.4	25.7±1.7	25.6±1.3	0.054	0.984
t_1_	20.5±2.1^*^	19.9±2.6^*^	19.7±2.0^*^	20.1±2.4^*^	0.595	0.619
t_2_	22.1±2.0^*&^	24.6±1.8^*#$^	21.3±2.2^*&^	23.7±2.1^*#$^	16.559	0.000
t_3_	24.7±1.9^*$&^	25.7±1.7^#$^	23.8±1.7^*#&^	24.7±1.7^*$&^	6.548	0.000
t_4_	25.2±1.6	25.6±2.0	25.4±1.9	25.6±2.1	0.346	0.792
t_5_	25.6±1.5	25.4±1.3	25.5±1.5	25.4±1.3	0.078	0.971
*F* values	50.719	47.233	56.648	45.495	—	—
*P* values	0.000	0.000	0.000	0.000	—	—

*MMSE* Mini-mental State Examination, *EN*_*1*_ Etomidate-remifentanil with duration of surgery less than 60min, *ED*_*1*_ Etomidate-remifentanil and dexmedetomidine with duration of surgery less than 60min, *EN*_*2*_ Etomidate-remifentanil with duration of surgery more than 60min, *ED*_*2*_ Etomidate-remifentanil and dexmedetomidine with duration of surgery more than 60min. t_0_ 30 minutes before anesthesia, t_1_ 3h after surgery, t_2_ 8h after surgery, t_3_ 24h after surgery, t_4_ 48h hours after surgery and t_5_ 72h after surgery. *p < 0.05 vs.t_0_, ^#^*p* < 0.05 vs.EN1, ^$^*p* < 0.05 vs.EN_2_ and ^&^*p* < 0.05 vs.ED_1_

The ZSDS scores at different time points in four groups are shown in Table 5. Compared to t_0_, the ZSDS scores at t_1-2_ in EN_1_, EN_2_ and ED_2_ groups and t_1_ in ED_1_ group were increased more significantly (*p* < 0.05). The ZSDS scores at t_1-2_ were higher in EN_1_ and EN_2_ groups than in ED_1_ and ED_2_ groups, respectively.

**Table 5:** The Zung Self-Rating Depression Scale scores at different time points in four groups

Time points	EN_1_ group *n*=34	ED_1_ group *n*=31	EN_2_ group *n*=32	ED_2_ group *n*=35	*F* values	*P* values
t_0_	42.7±2.8	42.2±3.0	43.1±3.1	42.1±2.8	0.917	0.435
t_1_	47.9±2.4^*^	44.5±2.7^*#$^	49.2±2.6^*^	45.1±2.4^*#$^	25.639	0.000
t_2_	46.7±2.4^*^	42.5±2.1^#$^	47.2±3.0^*^	43.7±2.9^*#$^	23.262	0.000
t_3_	42.6±2.2	41.6±2.3	42.3±2.3	41.5±3.1	1.391	0.248
t_4_	41.5±2.7	42.56±2.7	41.8±2.9	42.1±2.8	0.633	0.594
t_5_	42.3±2.5	41.8±2.1	43.1±2.7	41.6±2.4	2.199	0.091
*F* values	37.108	5.471	36.783	9.061	—	—
*P* values	0.000	0.000	0.000	0.000	—	—

*EN*_*1*_ Etomidate-remifentanil with duration of surgery less than 60min, *ED*_*1*_ Etomidate-remifentanil and dexmedetomidine with duration of surgery less than 60min, *EN*_*2*_ Etomidate-remifentanil with duration of surgery more than 60min, *ED*_*2*_ Etomidate-remifentanil and dexmedetomidine with duration of surgery more than 60min. t_0_ 30 minutes before anesthesia, t_1_ 3h after surgery, t_2_ 8h after surgery, t_3_ 24h after surgery, t_4_ 48h hours after surgery and t_5_ 72h after surgery. * *p* < 0.05 vs.t_0_, ^#^ *p* < 0.05 vs.EN_1_ and ^$^ *p* < 0.05 vs.EN_2_

The State-Trait Anxiety Inventory scores at different time points in four groups are shown in Table 6. Compared to t_0_, the S-AI scores at t_1-2_ in EN_1_, ED_1_and ED_2_ groups and t_1-3_ in EN_2_ group were increased more significantly (*p* < 0.05). Both the S-AI scores and the T-AI scores at t_1-2_ were higher in EN_1_ and EN_2_ groups than in ED_1_ and ED_2_ groups (*p* < 0.05), respectively. Compared to t_0_, the T-AI scores at t_1-2_ in EN_1_ and ED_1_ groups and t_1-3_ in EN_2_ and ED_2_ groups were increased more significantly (*p* < 0.05).

**Table 6:** The State-Trait Anxiety Inventory scores at different time points in four groups

Indexes	Groups	n	t_0_	t_1_	t_2_	t_3_	t_4_	t_5_	*F* values	*P* values
S-AI	EN1	34	39.7±2.2	47.5±3.0*	46.2±2.8*	40.7±2.7^$^	39.7±2.9	39.3±2.2	64.896	0.000
	ED1	31	40.2±3.0	44.5±2.5*^#$^	42.5±2.3*^#$^	40.1±1.8^$^	39.7±2.4	39.8±3.0	18.597	0.000
	EN2	32	39.8±2.8	48.3±2.7*	46.6±2.5*	43.3±2.4*^#^	39.8±2.5	40.1±2.3	66.797	0.000
	ED2	35	39.8±2.7	45.1±2.7*^#$^	43.1±2.4*^#$^	41.0±2.7^$^	39.8±2.4	40.0±2.6	21.721	0.000
	*F* values	0.206		14.260	21,320	10.689	0.023	0.727	——	
	*P* values	0.891		0.000	0.000	0.000	0.995	0.537	——	
T-AI	EN1	34	39.9±2.7	48.4±2.4*	47.1±3.1*	40.5±2.5	39.47±2.3	39.9±3.0	77.193	0.000
	ED1	31	39.6±3.0	45.6±3.7*^#$^	42.2±2.9*^#$^	40.7±3.2	39.8±2.8	39.5±3.1	17.807	0.000
	EN2	32	39.7±3.3	48.8±2.6*	47.6±2.4*	42.5±2.9*^#^	40.0±2.7	39.9±3.2	60.479	0.000
	ED2	35	39.4±3.0	46.5±3.8*^#$^	42.7±3.3*^#$^	41.6±2.9*	39.6±3.1	39.8±3.3	22.990	0.000
	*F* values	0.276		7.420	28.984	3.131	0289	0.297	——	
	*P* values	0.842		0.000	0.000	0.028	0833	0.827	——	

*EN*_*1*_ Etomidate-remifentanil with duration of surgery less than 60min, *ED*_*1*_ Etomidate-remifentanil and dexmedetomidine with duration of surgery less than 60min, *EN*_*2*_ Etomidate-remifentanil with duration of surgery more than 60min, *ED*_*2*_ Etomidate-remifentanil and dexmedetomidine with duration of surgery more than 60min. t_0_ 30 minutes before anesthesia, t_1_ 3h after surgery, t_2_ 8h after surgery, t_3_ 24h after surgery, t_4_ 48h hours after surgery and t_5_ 72h after surgery. *p < 0.05 vs.t_0_, ^#^ *p* < 0.05 vs.EN_1_ and ^$^ *p* < 0.05 vs.EN_2_

The recalled Arabic numbers at different time points in four groups are shown in Table 7. Compared to t_0_, the recalled Arabic numbers at t_1-3_ in EN_1_ and EN_2_ groups and t_1-2_ in ED_1_ and ED_2_ groups were decreased more significantly (*p* < 0.05). The recalled Arabic numbers at t_1-3_ were lower in EN_2_ groups than in EN_1_ group (*p* < 0.05). Compared to EN_2_ group, the recalled Arabic numbers at t_1-3_ were higher in ED_2_ group (*p* < 0.05).

**Table 7:** The recalled Arabic numbers at different time points in four groups

Time points	EN_1_ group *n*=34	ED_1_ group *n*=31	EN_2_ group n=32	ED_2_ group *n*=35	*F* values	*P* values
t0	4.8±0.4	4.7±0.5	4.6±.0.5	4.6±0.7	1165	0.326
t1	2.4±0.7*^$^	2.6±0.7*^$^	1.6±0.6*^#&^	2.3±0.6*^$&^	25.639	0.000
t2	3.1±0.8*^$^	3.4±0.7*^$^	2.4±0.5*^#&^	3.1±0.9*^$&^	23.262	0.000
t3	4.2±0.8*^$^	4.4±0.8^$^	3.4±0.8*^#&^	4.2±0.8^$^	1.391	0.248
t4	4.5±0.7	4.5±0.5	4.4±0.7	4.3±0.7	0.633	0.594
t5	4.6±0.5	4.5±0.6	4.6±0.5	4.4±0.7	2.199	0.091
*F* values	70.829	48.195	123.069	59.942	—	—
*P* values	0.000	0.000	0.000	0.000	—	—

*EN*_*1*_ Etomidate-remifentanil with duration of surgery less than 60min, *ED*_*1*_ Etomidate-remifentanil and dexmedetomidine with duration of surgery less than 60min, *EN*_*2*_ Etomidate-remifentanil with duration of surgery more than 60min, *ED*_*2*_ Etomidate-remifentanil and dexmedetomidine with duration of surgery more than 60min. t_0_ 30 minutes before anesthesia, t_1_ 3h after surgery, t_2_ 8h after surgery, t_3_ 24h after surgery, t_4_ 48h hours after surgery and t_5_ 72h after surgery. *p < 0.05 vs.t_0_, ^#^*p* < 0.05 vs.EN_1_, ^$^*p* < 0.05 vs.EN_2_ and ^&^*p* < 0.05 vs.ED_1_

The plasma concentrations of S-100β at different time points in four groups are shown in Table 8. Compared to t_0_, the plasma concentration of S-100β at t_1-2_ in EN_1_ and ED_1_ groups and at t_1-3_ in EN_2_ and ED_2_ groups were increased more significantly (*p* < 0.05). The plasma concentrations of S-100β at t_1-2_ in EN_2_ group and t_1-3_ in ED_2_ group were increased more significantly than in EN_1_ and ED_1_ groups (*p* < 0.05), respectively. The plasma concentration of S-100β at t_1-2_ in EN_1_ group and t_1-3_ in EN_2_ group were higher than in ED_1_ and ED_2_ groups (*p* < 0.05), respectively.

**Table 8:** The plasma concentration of S-100β at different time points in four groups

Time points	EN_1_ group( pg/ml) *n*=34	ED_1_ group( pg/ml) *n*=31	EN_2_ group( pg/ml) *n*=32	ED_2_ group( pg/ml) *n*=35	*F* values	*P* values
t_0_	66.5±7.3	66.8±9.0	66.9±0.6	66.8±7.5	0.0217	0.995
t_1_	121.0±17.4*^$^	108.6±12.9*^#$^	143.6±13.9*^#^	125.5±15.6*^$&^	28.391	0.000
t_2_	105.6±11.9*^$^	83.1±7.9*^#$^	117.4±13.1*^#^	107.0±18.0*^$&^	37.2856	0.000
t_3_	69.0±8^$^	67.3±8.7^$^	94.8±10.8*^#^	81.3±16.7*^#$&^	39.438	0.000
t_4_	67.5±6.7	67.0±9.0	70.7±8.1	67.5±6.8	1.574	0.198
t_5_	66.9±7.5	66.8±9.4	67.3±7.6	66.6±7.1	0.036	0.991
*F* values	178.513	94.065	286.857	130.356	—	—
*P* values	0.000	0.000	0.000	0.000	—	—

*S-100β* S-100 calcium-binding protein B, *EN*_*1*_ Etomidate-remifentanil with duration of surgery less than 60min, *ED*_*1*_ Etomidate-remifentanil and dexmedetomidine with duration of surgery less than 60min, *EN*_*2*_ Etomidate-remifentanil with duration of surgery more than 60min, *ED*_*2*_ Etomidate-remifentanil and dexmedetomidine with duration of surgery more than 60min. t_0_ 30 minutes before anesthesia, t_1_ 3h after surgery, t_2_ 8h after surgery, t_3_ 24h after surgery, t_4_ 48h hours after surgery and t_5_ 72h after surgery. * *p* < 0.05 vs.t_0_, ^#^ *p* < 0.05 vs.EN_1_, ^$^ *p* < 0.05 vs.EN_2_ and ^&^*p* < 0.05 vs.ED_1_

The plasma concentrations of NSE at different time points in four groups are shown in Table 9. Compared to t_0_, the plasma concentration of NSE at t_1-3_ in EN_1_, ED_1_ and ED_2_ groups and t_1-4_ in EN_2_ group were increased more significantly (*p* < 0.05). The plasma concentration of NSE at t_1-2_ and t_4_ were increased more significantly in EN_2_ group than in EN_1_ group (*p* < 0.05). The plasma concentrations of NSE at t_1-3_ were higher in EN_1_ group than in ED_1_ group (*p* < 0.05). The plasma concentrations of NSE at t_1-4_ were decreased more significantly in ED_2_ group compared to EN_2_ group (*p* < 0.05).

**Table 9:** The plasma concentration of NSE at different time points in four groups

Time points	EN_1_ group (ng/ml) *n*=34	ED_1_ group (ng/ml) *n*=31	EN_2_ group (ng/ml) *n*=32	ED_2_ group (ng/ml) *n*=35	*F* values	*P* values
t_0_	17.6±1.3	17.8±1.2	17.7±1.3	17.9±1.3	0.139	0.935
t_1_	45.3±8.1*^$&^	38.3±6.7*^#$^	51.2±6.8*^#&^	42.8±7.1*^$&^	18.370	0.000
t_2_	37.1±6.9*^$&^	28.2±5.9*^#$^	40.7±5.6*^#&^	35.1±7.0*^$&^	21.853	0.000
t_3_	28.9±6.3*^&^	18.3±1.4*^#$^	31.1±4.2*^&^	26.6±6.3*^#$&^	38.302	0.000
t_4_	18.2±1.4^$^	17.9±1.2^$^	24.9±3.4*^#&^	18.3±1.7^$^	84.180	0.000
t_5_	17.4±1.3	17.7±1.3	17.5±1.4	17.7±1.12	0.324	0.807
*F* values	178.437	179.000	152.111	306.952	—	—
*P* values	0.000	0.000	0.000	0.000	—	—

*NSE* Neuron-specific enolase, *EN*_*1*_ Etomidate-remifentanil with duration of surgery less than 60min, *ED*_*1*_ Etomidate-remifentanil and dexmedetomidine with duration of surgery less than 60min, *EN*_*2*_ Etomidate-remifentanil with duration of surgery more than 60min, *ED*_*2*_ Etomidate-remifentanil and dexmedetomidine with duration of surgery more than 60min. t_0_ 30 minutes before anesthesia, t_1_ 3h after surgery, t_2_ 8h after surgery, t_3_ 24h after surgery, t_4_ 48h hours after surgery and t_5_ 72h after surgery. *p < 0.05 vs.t_0_, ^#^ *p* < 0.05 vs.EN_1_, ^$^ *p* < 0.05 vs.EN_2_ and ^&^ *p* < 0.05 vs.ED_1_

## Discussion

In present study, we found that cognitive dysfunction, as well as depression and anxiety were developed in older patients undergoing total intravenous anaesthesia with etomidate and remifentanil within 8-24h after surgery. The cognitive dysfunction was more severe in the long-term anesthesia group, while there was no difference in the severity of anxiety and depression between the short-term and long-term anesthesia groups. The plasma concentrations of S-100β and NSE were increased significantly within 3-48h after surgery. The administration of dexmedetomidine could improve postoperative cognitive dysfunction, emergence agitation, depression and anxiety, attenuate the plasma concentrations of S-100β and NSE and reduce the dosage of etomidate and remifentanil administered during the operation.

Older patients are more likely to develop postoperative cognitive dysfunction (POCD) after surgery and anesthesia [10]. The incidence of POCD in older patients ( > 60 years old ) is about 25.8 % within 7 days after surgery and 10 % within 3 months after surgery. In our study, the older patients developed POCD 1-2d after surgery, which was consistent with our previous findings [11]. It is suggested that POCD in older patients mainly occurs in a short time after surgery. Lower educational level, advancing age, type of surgery, intraoperative hypotension, anaesthetic methods, duration and depth of anaesthesia, anaesthetics and postoperative pain management have been suggested to be potentially correlated with risk of POCD [12]. Etomidate has been advocated clinically for anaesthesia in older and critically ill patients for its good intraoperative hemodynamic stability [13]. Intraoperative hypotension is closely related to postoperative cognitive dysfunction. Compared with propofol, screening colonoscopy with etomidate had lower incidence of hypotension in patients of all ages [14]. Therefore, cognitive dysfunction in patients with etomidate anesthesia should be improved compared with patients with propofol anesthesia. However, compared with our previous clinical study [11], older patients with propofol or etomidate anesthesia all experienced transient (within 24 hours) cognitive dysfunction after surgery. It may be that the adverse effects of etomidate itself on POCD attenuated the improvements of intraoperative hemodynamic stability on POCD in older patients [15]. Animal studies have shown that etomidate induced long-term memory loss and cognitive dysfunction [15, 16]. In our study, we found that both the memory ability of Arabic numerals and cognitive function were impaired in older patients anaesthetized with etomidate, which was consistent with the above findings. The Neuron specific enolase (NSE) and s100 β Proteins are biomarkers of brain injury, which are positively correlated with POCD [17]. A clinical study found that the scores of Paired Associate Learning test and Benton Visual Retention Test were decreased and the plasma concentration of S100B was increased one week postoperatively [18]. In present study, the plasma concentrations of S-100β and NSE were increased significantly 3-48h after surgery, while the MMSE scores were decreased significantly 3-24h postoperatively. It indicated that the increase of plasma S100B and NSE levels were related to the development of POCD [19]. Kavrut et al. reported that the duration of anesthesia in patients with POCD at 7 days and 3 months after surgery was significantly longer than that in patients without POCD [19]. In the present study, the MMSE scores at 24h after surgery were decreased more significantly in patients with long-term surgery compared with patients with short-term surgery. It was suggested that the increasing duration of surgery would aggravate the incidence and severity of POCD [20].

A meta-analysis showed that the intraoperative administration of dexmedetomidine could effectively reduce the incidence of POCD, and improve the MMSE scores on the first, third and seventh postoperative days in patients undergoing general anesthesia [21]. Another meta-analysis showed that dexmedetomidine was related to the reduced risk of POCD in older patients under general anesthesia, and dexmedetomidine was recommended as a preventive measure for postoperative cognitive dysfunction in older patients [22]. The present study showed that the intravenous infusion of dexmedetomidine could attenuate the decrease of MMSE scores in older patients, which was consistent with the results of Patel et al [23]. The dosages of etomidate both in short-term and long–term surgery groups were reduced by the administration of dexmedetomidine during surgery in our study. The activation of microglia induced by the administration of etomidate created an inflammatory environment and stimulated A1-specific astrocyte response, which induced persistent synaptic inhibition and cognitive deficits [15]. Cho et al. [24] reported that the administration of dexmedetomidine improved the cognitive impairment and reduced pro-inflammatory cytokines by regulating ASC and NLRP3 expression in the POCD model. In our study, after administration of dexmedetomidine, the decrease of etomidate dosage during surgery attenuated the postoperative memory loss and cognitive dysfunction induced by etomidate and central nervous system inflammation was inhibited, which led to the improvement of POCD in older patients under etomidate anesthesia.

Clinical study reported that both anxiety and depression levels were increased significantly following surgery [25]. This is mainly due to the patient's estimation of pain and the risk of death, which leads to fear of surgery and has a negative impact on their psychological condition. In addition, they are separated from their professional life, friends, and family before and after surgery. The inability to adapt to this situation can lead to increased anxiety and depression [26]. At present, the results of studies on the relationship between postoperative cognitive dysfunction and perioperative depression and anxiety in older patients are inconsistent. Wang et al. found that perioperative depression and anxiety had no effect on cognitive function of older patients [27]. However, Oyeyemi et al. reported that poorer perioperative cognitive function was associated with greater severity of anxiety and depression in older patients [28]. Depression after cardiac surgery could lead to cognitive impairment [29]. In our previous [11] and present studies, the ZSDS scores at 3-8h and STAI scores at 3-24h after surgery were increased significantly, while the MMSE scores at 3-24h were decreased. It was suggested that postoperative anxiety and depression might exacerbate the POCD in older patients. The scores of hospital anxiety and depression scale and anxiety subscale on the second day after delivery in dexmedetomidine group were significantly better than those in non- dexmedetomidine group [30]. In our study, the ZSDS scores at 3-8h and STAI scores at 3-24h after surgery were lower in ED_1_ and ED_2_ groups than in EN_1_ and EN_2_ groups, which was consistent with the above study of Wang et al. [30] Moreover, the MMSE scores at 8-24h after surgery were higher in ED_1_ and ED_2_ groups than in EN_1_ and EN_2_ groups, and the plasma concentration of S-100β and NSE were lower in ED_1_ and ED_2_ groups than in EN_1_ and EN_2_ groups 3-24h after surgery, respectively. This finding indicated that intravenous infusion of dexmedetomidine could improve the POCD by reducing the severity of anxiety and depression in older patients.

Emergence agitation (EA) often occurs during the initial 30 - 60 min in recovery from general anesthesia, which was characterized as disorientation, confusion, hallucinations and agitation during emergence from general anesthesia [31]. The intraoperative administration of low-dose dexmedetomidine could reduce emergence agitation and stabilize perioperative haemodynamics [32]. The RSAS scores at 5, 15, 30 and 60min after extubation were higher in EN_1_ and EN_2_ groups than in ED_1_ and ED_2_ groups in present study, suggesting that intraoperative intravenous dexmedetomidine reduced the EA, which was consistent with the results of Sun et al. [32]. After intravenous dexmedetomidine in older patients, the recovery time of anesthesia, extubation time and PACU stay time were delayed in our study. In contrast, Sun et al. reported that intraoperative administration of dexmedetomidine had no effects on the recovery time of anesthesia and extubation time [32]. The dose of intraoperative dexmedetomidine administered might be responsible for the differences. The dose of intravenous dexmedetomidine was 0.2μg·kg^-1^·h^-1^ in study of Sun et al., and 0.4μg·kg^-1^·h^-1^ in our study. We did not investigate the effects of low-dose dexmedetomidine on cognitive function in older patients in present study. Future studies should explore the optimal dose of dexmedetomidine to improve POCD in older patients without delaying the recovery of anesthesia.

This study has several limitations. Firstly, Liu et al. [16] reported that the inhibition of hippocampal glial cells induced by etomidate could inhibit neuronal activity and trigger memory loss. The administration of dexmedetomidine improved the Arabic digital memory ability in present study. It is unclear whether dexmedetomidine affects the inhibitory effect of etomidate on hippocampal astrocytes. Secondly, we simply divided patients into two groups based on the surgery time and observed the effect of the surgery time on the occurrence of POCD in older patients. The effects of different surgery time on both POCD induced by etomidate and dexmedetomidine improving POCD in older patients under etomidate anesthesia should be explored in the future. Thirdly, Sun et al. reported that intraoperative administration of dexmedetomidine 0.2μg·kg^-1^·h^-1^ had no effects on the recovery time of anesthesia and extubation time [29]. In our study, anesthesia recovery time and extubation time were delayed in older patients receiving intravenous dexmedetomidine 0.4μg·kg^-1^·h^-1^. Next, we will investigate the optimal dose of dexmedetomidine to improve POCD in older patients without delaying the recovery of anesthesia and extubation time. Fourthly, in our study, given that the surgery caused less trauma to the older patients, we only observed the occurrence of POCD in patients within 3 days after surgery. The effects of intravenous dexmedetomidine on postoperative long-term cognitive function in older patients anesthetized with etomidate were unclear.

In conclusion, cognitive dysfunction, as well as depression and anxiety were developed in older patients undergoing total intravenous anaesthesia with etomidate within 8-24h after surgery. The administration of dexmedetomidine could improve postoperative cognitive dysfunction, emergence agitation, depression and anxiety, attenuate the plasma concentrations of S-100β and NSE and reduce the dosage of etomidate and remifentanil administered during surgery.

## Data Availability

The datasets used and/or analysed during the current study available from the corresponding author on reasonable request.
